# Lineage switch of B‐acute lymphoblastic leukemia with t(11;19)(q23;p13.3) into acute myeloid leukemia with monocytic differentiation after induction chemotherapy

**DOI:** 10.1002/jha2.679

**Published:** 2023-05-01

**Authors:** Katelynn M. Wilton, Gang Zheng

**Affiliations:** ^1^ Mayo Clinic Medical Scientist Training Program Rochester Minnesota USA; ^2^ Department of Pathology and Laboratory Medicine Mayo Clinic Rochester Minnesota USA

1

A 65‐year old woman with dyspnea, headaches, and epistaxis presented with anemia (hemoglobin 7.7 g/dL) and thrombocytopenia (platelets 18×10^9^/L) with a white blood cell (WBC) count of 4.0 × 10^9^/L and 60% circulating blasts. Bone marrow blast percentage was 95%, blasts were small to intermediate in size with reticulated chromatin and scant cytoplasm on bone marrow aspirate (Figure 1, panel A, original magnification 1000×, Wright–Giemsa stain) and biopsy (Figure 1, panel B, original magnification 600×, hematoxylin and eosin stain). The leukemic cells had a B cell (CD19^+^CD20^−^CD22^+^cCD79a^+^) immunophenotype without markers of immaturity (CD34^−^Tdt^−^ CD10^−^) or myeloid lineage (MPO^−^CD33^−^CD13^−^, Figure 1, panel C). Cytogenetic study showed t(11;19)(q23;p13.3) (Figure 1, panel D), and fluorescence in situ hybridization confirmed KMT2A::MLLT1 fusion (nuc ish(MLL,MLLT1)x3(MLL con MLLT1×2)[479/500]). After one cycle of hyper‐CVAD chemotherapy, she remained anemic (hemoglobin 7.6 g/dL) with improved thrombocytopenia (platelets 169 × 10^9^/L) with a WBC count of 2.1 × 10^9^/L. A subsequent bone marrow aspirate (Figure 1, panel E, original magnification 1000×, Wright–Giemsa stain) and biopsy (Figure 1, panel F, original magnification 600×, hematoxylin and eosin stain) showed acute leukemia with monocytic differentiation with 71% monoblasts and promonocytes. Leukemic cells expressed CD33 and lysozyme with variable retained PAX5 (Figure 1, panel G, original magnification 200×). The karyotype of t(11;19)(q23;p13.3) confirmed persistent disease with lineage switch. The patient then received Venetoclax combined with FLAG‐IDA (fludarabine, cytarabine, idarubicin, and granulocyte colony‐stimulating factor) induction, and achieved complete remission. The next step of the treatment plan will be matched, unrelated donor allogeneic stem cell transplant.

**FIGURE 1:**
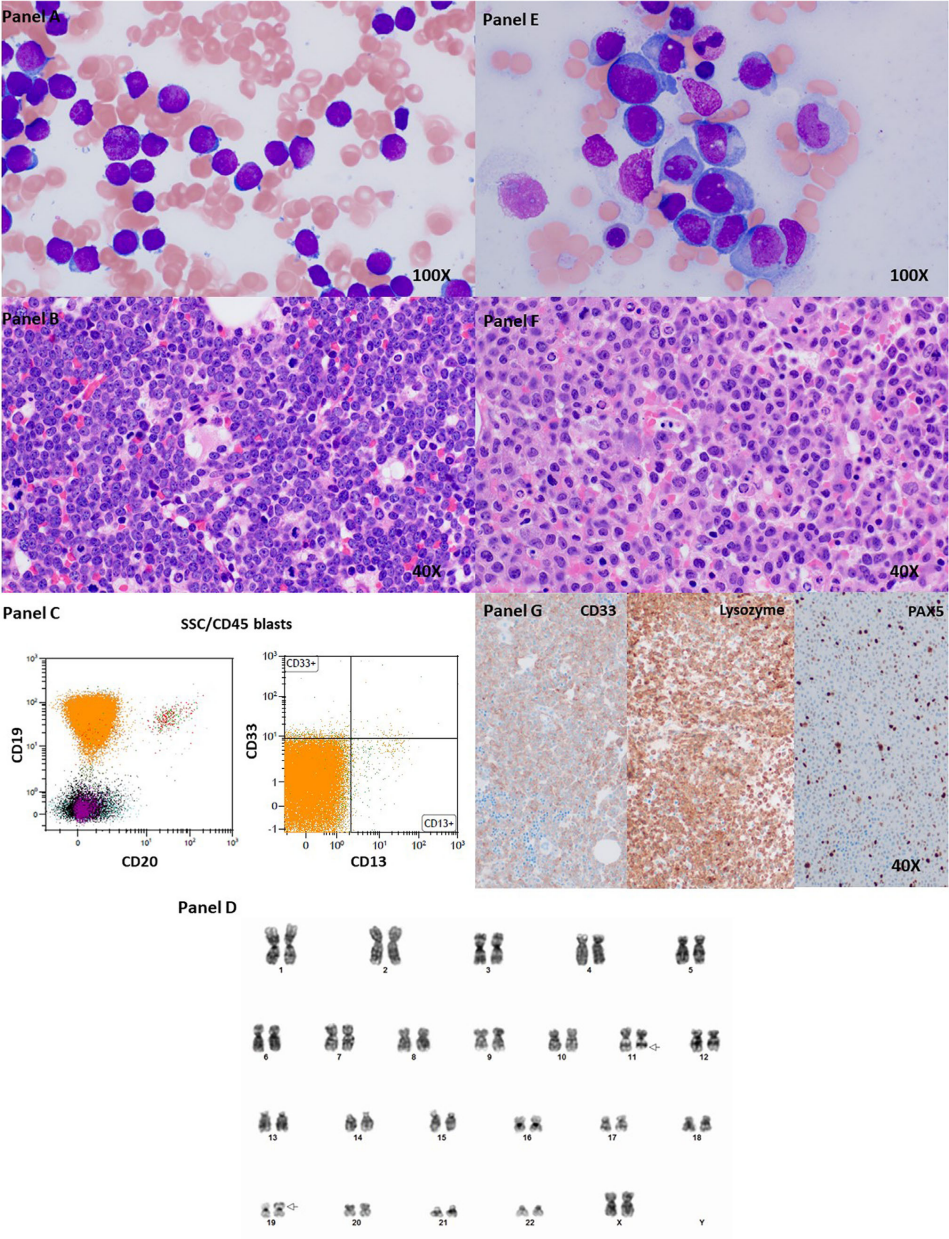
Lineage switch of B‐acute lymphoblastic leukemia with t(11;19)(q23;p13.3) into acute myeloid leukemia with monocytic differentiation.

Lineage switch of acute leukemia, including B‐ALL with t(11;19)(q23;p13.3), has been reported in pediatric patients (PMID: 22685031; 35409391) but is rare in adult patients, often occurs after or even during induction chemotherapy, and confers a poor prognosis. Most such acute leukemia harbor MLL (KMT2A) gene rearrangements.

## CONFLICT OF INTEREST STATEMENT

The authors declare no conflict of interest.

